# The Regulation of the Disease-Causing Gene *FXN*

**DOI:** 10.3390/cells13121040

**Published:** 2024-06-15

**Authors:** Yi Na Dong, Elizabeth Mercado-Ayón, Jennifer Coulman, Liam Flatley, Lucie Vanessa Ngaba, Miniat W. Adeshina, David R. Lynch

**Affiliations:** 1 Departments of Pediatrics and Neurology, The Children’s Hospital of Philadelphia, Philadelphia, PA 19104, USA; 2 Perelman School of Medicine, University of Pennsylvania, Philadelphia, PA 19104, USA; 3 The Wharton School, University of Pennsylvania, Philadelphia, PA 19104, USA

**Keywords:** *FXN*, Friedreich’s ataxia, GAA repeat expansion, transcription factors, iron, miRNAs, chaperones, proteasome, mitochondrial proteases, autophagy

## Abstract

Friedreich’s ataxia (FRDA) is a progressive neurodegenerative disease caused in almost all patients by expanded guanine–adenine–adenine (GAA) trinucleotide repeats within intron 1 of the *FXN* gene. This results in a relative deficiency of frataxin, a small nucleus-encoded mitochondrial protein crucial for iron–sulfur cluster biogenesis. Currently, there is only one medication, omaveloxolone, available for FRDA patients, and it is limited to patients 16 years of age and older. This necessitates the development of new medications. Frataxin restoration is one of the main strategies in potential treatment options as it addresses the root cause of the disease. Comprehending the control of frataxin at the transcriptional, post-transcriptional, and post-translational stages could offer potential therapeutic approaches for addressing the illness. This review aims to provide a general overview of the regulation of frataxin and its implications for a possible therapeutic treatment of FRDA.

## 1. Introduction

Friedreich’s ataxia (FRDA) is the most common hereditary ataxia, with an incidence of 1 in 50,000 people in Caucasian populations worldwide. FRDA is characterized by progressive gait and limb ataxia, scoliosis, dysarthria, visual loss, and hypertrophic cardiomyopathy [1]. The pathological change appears to occur first in the large sensory neurons of the dorsal root ganglia (DRG) and their axons in the posterior columns, with later atrophy of the corticospinal and spinocerebellar tracts of the spinal cord and the dentate nucleus in the cerebellum [2,3,4]. The age of onset is usually within the first two decades of life, and individuals are wheelchair-bound within 10–15 years of disease onset [5,6]. For patients with FRDA, cardiomyopathy-associated heart failure is the main cause of mortality.

FRDA is usually (96%) caused by biallelic guanine–adenine–adenine (GAA) trinucleotide repeats within intron 1 of the *FXN* gene [7]. The expanded GAA repeats transcriptionally repress *FXN* gene expression, leading to reduced frataxin proteins. Frataxin is involved in iron homeostasis [8,9], the biosynthesis of iron–sulfur clusters (ISCs) [10,11,12], and energy production in the cell [13,14]. Frataxin is a mitochondrial matrix localized protein that is encoded in the nucleus. It is synthesized as a precursor in the cytosol that is imported into the mitochondria, where it undergoes a sequential cleavage to mature into a functional frataxin [15]. A lack of frataxin leads to altered iron metabolism, decreased energy production, and increased oxidative stress, all of which ultimately result in pathological changes. Frataxin levels in peripheral tissues correlate with both the disease severity and the age of onset [16]. Since FRDA is caused by a deficiency in frataxin, which is controlled at transcriptional, post-transcriptional, and post-translational levels, several strategies are being developed to restore frataxin levels toward normal in order to treat the disease, including gene therapy, gene editing, and interventions to slow down the turnover of frataxin protein. The components of frataxin regulation will be outlined in this review along with their treatment-related implications.

## 2. Transcriptional Regulation of the *FXN* Gene

Transcriptional regulation is a crucial regulatory mechanism that governs the expression of the *FXN* gene. Multiple factors, including the size of the GAA repeats within the pathological range, transcription factors, and activators, have been identified to influence the transcription of *FXN* gene, both physiologically and pathologically. These factors are also the focus of therapy directed towards frataxin restoration.

## 3. *FXN* Gene Structure

The *FXN* gene is encoded in the long arm(q) of chromosome 9 (9q13–q21) and spans 150 kb [17,18]. Its promoter is not a typical TATA box, but instead, a CpG promoter with CpG island in the vicinity of the transcription start site [7]. The main transcription start site is 220 bp upstream from the start codon, and it includes an E-box/Mt-binding site [19]. Transcription factors that bind to this site and enhance *FXN* expression include SRF and TFAP2 [20]. The *FXN* gene contains six exons (1–4, 5a, and 6) [7]. Exon 1, which encodes an N-terminal fragment (55 amino acids) including the mitochondrial targeting sequence, is followed by intron 1, which contains 10,436 bp including the GAA repeats [19,21]. A total of 96% of FRDA patients have biallelic (100–1700) GAA triplet repeat expansions; 4% have GAA expansion in one allele and point mutation or deletion in the other *FXN* allele [7]. The repeat starts ~1 kb from the start of intron 1 [19]. Given that some epigenetic marks including acetylation of histone at lysine 27 (H3K27ac) and methylation of histone H3 at lysine 4 (H3K4me1,2,3) typically present in regulatory/enhancer regions are found in parts of exon 1 and intron 1, those regions are likely needed for *FXN* transcription. More precisely, the first 110 bp endogenous sequence in intron 1 are indispensable for frataxin expression [22]. In addition, at the 5′UTR before the start codon, 115 bp are necessary for *FXN* transcription [22]. This sequence may also be a binding region for the transcription factor TFAP2, which is critical for frataxin expression [22].

## 4. *FXN* Transcript Isoforms

Four different *FXN* gene transcripts have been identified. *FXN-1* mRNA is composed of six exons (1–4, 5a, and 6) and encodes a 210-amino acid protein, the canonical frataxin isoform (also called FXN-M) ubiquitously expressed in all tissues [23]. Instead of exon 5a, *FXN-3* mRNA has exon 5b. Exon 5b has an in-frame stop codon, so that *FXN-3* transcript generates a shorter 171 amino acid protein, whose 11 COOH-terminal residuals differ from *FXN-1* [7]. *FXN-2* mRNA was found in an attempt to clone the full-length frataxin cDNA by PCR targeting the extremities of the *FXN-1* coding sequence. *FXN-2* has an 8 bp insertion between exons 4 and 5a due to an alternative splicing site at the 5′ end of intron 4. The 8 bp insertion generates a frameshift that introduces a new stop codon site. The *FXN-2* transcript thus encodes a 196-amino-acid protein that differs from FXN-1 after residue 160. This transcript is found at lower levels in brain, cerebellum, spinal cord, heart, and skeletal muscle [24]. In human mononuclear cells, *FXN-2* and *FXN-3* mRNA expressions are 4.72% and 2.47% of *FXN-1* mRNA, respectively. FXN-2 and FXN-3 not only have identical residues in the functional region (amino acids 90–160) compared with FXN-1 but also have the same ability to interact with synthetic enzymes for iron–sulphur complexes, suggesting a possible biological role of FXN-2 and FXN-3 [25]. To support this, the antioxidant tocotrienol, a member of the vitamin E family, preferentially increases *FXN-3* mRNA in FRDA patients. Neither FXN-1 nor FXN-2 shows any effects [25]. The underlying mechanism may involve variable splicing regulation in addition to an increase in gene transcription and/or mRNA half-life.

*FXN-4* mRNA, also called *FXN-E*, is a novel isoform lacking the mitochondrial targeting sequence. *FXN-E* transcript originates in intron 1 via non-coding exon 1b, which independently splices to exon 2 via three alternate splice donor sites (IIa, IIb, and IIc) [26]. The predicted translational initiation codon for all variants of *FXN-E* is located within exon 2 and corresponds to the methionine at position 76 in FXN-1. Thus, this transcript encodes a 135-amino-acid protein (76–210) with an acetylated N-Terminus. FXN-E is found at relatively high levels in erythrocytes [27], and is extramitochondrial because it does not possess a mitochondrial targeting sequence. FXN-E is also expressed in the cerebellum and heart from both humans and animals [26]. Blood samples from typical FRDA patients show lower levels of this protein, which correlate with the length of shorter enlarged GAA triplet-repeat in these patients. Similar to FXN-1, FXN-E modulates mitochondrial dynamics and function [23,28]. In individuals with a single expanded GAA allele and a point mutation in the early part of exon 1, FXN-E levels are normal in blood as the alternatively spliced exon is not affected. Such patients still have severe disease, showing that in vivo FXN-E cannot substitute for deficient FXN-M.

## 5. GAA Repeat Expansion

The GAA repeat expansion is the most significant factor affecting *FXN* gene transcription when compared to all other variables. Normal individuals have no more than 30 GAA repeats (and usually have around 7 repeats), whereas FRDA patients can have as many as 1700 GAA repeats. The length of the shorter of the two expanded alleles inversely correlates with FXN levels and age of onset and positively with disease severity [1]. FRDA-associated expanded GAA repeats originate from normal alleles by recurrent expansions of alleles at risk [29]. The size threshold that determines GAA repeat instability and expansion is between 26 and 44 uninterrupted GAA repeats [29,30,31]. Expanded GAA repeats are genetically unstable, exhibiting both expansions and contractions with a significant predilection for large contractions [31,32,33,34]. The somatic instability of expanded GAA repeats is length-dependent and tissue-specific, with significantly longer GAA tracts detected in hearts and pancreases than in other tissues [31,32,33]. The expansion bias found in hearts and pancreases is likely to contribute to the onset of symptoms and disease progression. While maternally transmitted expansions can contract or expand with equal frequency, paternal transmission typically results in a contraction of the repeats [29,35,36,37].

The pathogenic nature of expanded GAA repeats is determined by its unusual DNA structure. Uninterrupted long GAA repeats adopt an intramolecular R·R·Y triplex structure resulting in length- and orientation-dependent transcriptional inhibition both in vitro [38] and in vivo [39,40]. On the other hand, intronic interrupted GAA repeats, like hexanucleotide repeat (GAAGGA)_65_, do not inhibit transcription like same-length GAA repeats do, are not associated with FRDA, and can be stably transmitted from parent to child for three siblings [40]. Short interruptions like (GAGGAA)_5–9_ are also found in normal individuals and appear to be nonpathogenic [29]. The structural analysis of hexanucleotide repeat (GAAGGA)_65_ demonstrates that it does not adopt a triplex conformation the way GAA repeats of similar length do, suggesting that the presence of a triplex structure is essential for the pathogenicity of expanded GAA repeat and its ability to suppress gene expression [40]. The greater the extent of interruptions, the less inhibition of in vitro transcription [41]. FRDA patients with small interruptions at the 3′ end of the GAA repeat tract are linked to shorter GAA1 repeat tracts and a later age at disease onset, which is consistent with the impact of interruptions on *FXN* gene transcription [42]. Large interruptions are extremely uncommon in the expanded GAA repeats of FRDA [43].

GAA repeat expansion not only adopts a triplex structure but also forms hybrid conformations between DNA and RNA (R-loops) and heterochromatin to reduce *FXN* mRNA transcription [44,45,46,47,48]. Stable triplexes and R-loops impede RNA transcription on the *FXN* gene by either directly interfering with RNA polymerase (Pol) II transcription or sequestering transcription factors/RNA polymerase [45,46,47]. Heterochromatin-mediated transcriptional silencing is associated with epigenetic modifications in the intron 1 region flanking the GAA repeat expansion. Repressive histone marks, including histone trimethylation (H3K9me3 and H3K27me3) and hypoacetylation (H3 and H4), and DNA hypermethylation are among these alterations [19,26,49,50,51]. Both histone marks and DNA methylation are found in FRDA-patient-derived cells including brain tissue [26,51]. DNA methylation also correlates with *FXN* transcriptional deficiency and age of onset [26]. The chemical reactivation of transcription of the *FXN* gene with HDAC inhibitors or other chromatin targeting drugs can partially rescue frataxin deficiency [50,52]. There is also an interplay between R-loops and epigenetic changes. Increasing R-loop levels by treatment with the DNA topoisomerase inhibitor camptothecin upregulates repressive histone marks (H3K9me2), while a decrease in the amount of repressive histone mark has no effect on R-loop levels [45], suggesting that epigenetic changes are secondary to abnormal DNA confirmation.

Both transcriptional initiation and elongation are proposed to be involved in *FXN* gene transcriptional silencing [53,54,55]. In induced pluripotent stem cells (iPSCs) derived from FRDA fibroblasts, GAA repeat expansions have no effect on the recruitment of RNA Pol II to the *FXN* promoter region but significantly reduce the phosphorylation of Pol II at Serine 5 and Serine 2 at the C-terminal domain, an indicator of active initiation/elongation, suggesting the inhibitory effect of GAA repeat expansions on the transition from initiation to productive elongation [56]. Both FRDA and control cells can actively initiate *FXN* transcription; however, the GAA repeat expansion induces an aberrant transcription termination, resulting in a short and polyadenylated mRNA transcript that prematurely terminates upstream from the GAAs. This RNA transcript contains exon 1 and a fragment of the *FXN* intron1 and is alternatively spliced as the intronic part of this transcript lacks the first 683 nt, immediately downstream from the end of exon 1 (designated as *FXN-ett*, *FXN-5 mRNA*). Interestingly, *FXN*-ett level correlates with the length of the longer of the two GAA alleles and is stable and expressed in different cells, including FRDA patient cardiac cells and in FRDA-humanized transgenic mice. CRISPR-Cas9 excision of the expanded GAAs decreases *FXN-ett* expression and restores FXN expression, confirming that the GAA expansion is responsible for the transcriptional impediment during early elongation and formation of the aberrantly spliced, prematurely terminated *FXN-ett* RNA [56].

The profound impact of GAA repeat expansion on the transcriptional barrier of the *FXN* gene has led to the development of multiple strategies for their excision in an effort to restore normal amounts of *FXN* transcripts. CRISPR technology uses guide RNAs to identify the target sequence and Cas9 nuclease to break it. CRISPR-Cas9 efficiently removes the GAA repeat expansion from intron 1 in FRDA patient hematopoietic stem and progenitor cells, thus leading to increased frataxin expression and mitochondrial function [57]. An extension of that study also demonstrates improvement in cellular apoptosis and mitochondria–endoplasmic reticulum interactions in patient-derived iPSC neurons [58]. CRISPR-Cas9 can also remove the entire intron 1 in dorsal root ganglia organoids derived from FRDA patient iPSCs. This intron 1 excision reactivates *FXN* gene expression; reduces epigenetic silencing marks, such as H3K9me3 and H3K9ac at the intron 1 chromatin; and improves mitochondrial morphology in the DRG axons [59]. Interestingly, the deletion of most of intron does not impact frataxin expression despite many studies concluding that intron 1 contains transcriptional regulation sequences; perhaps, those sequences exist in mice but not human frataxin [59]. Another approach to increasing frataxin expression focuses on genomic editing with zinc finger nucleases, which removes one copy of the GAA repeat region from intron 1. This approach increases frataxin expression and reverses the biochemical phenotype associated with frataxin deficiency in cells [60].

## 6. Transcription Factors

The transcription factors identified for the *FXN* gene include serum response factor (SRF), transcription factor family activator protein 2 (TFAP2), and Octamer transcription factor-1 (Oct-1). SRF is a member of the MADS (MCM1, Agamous, Deficiens, and SRF) box superfamily of transcription factors, which binds to the serum response element (SRE) in the promotor region of target genes and participates in cell cycle regulation, cell growth, and differentiation [61,62]. TFAP2 is a developmentally regulated, retinoic-acid-inducible transcriptional activator [63], while Oct-1 binds to the “ATTTGCAT” sequence and regulates a variety of tissue-specific and general housekeeping genes [64,65]. Both SRF and TFAP2 bind to the region about 200 bp upstream from the start codon of the *FXN* gene while the binding site of Oct-1 is located 4.95 kb from the start codon [20,66]. The deletion of these transcription factors or mutations in the binding sites of these transcription factors result in a reduced expression of the *FXN* gene, while overexpressing SRF and TFAP2 increases the *FXN* mRNA levels in both cell lines and FRDA patient lymphoblasts [20]. Interestingly, the intronic sequence downstream from exon 1 is also necessary for SRF and TFAP2 activity. The transcriptional activity of SRF and TFAP2 is dramatically reduced upon removal of the intronic region downstream from exon 1. Both SRF and TFAP2 mRNA levels are decreased in FRDA patient lymphoblasts [20]. TFAP2 mRNA levels are also subject to iron regulation. Iron depletion with iron chelator DFO decreases TFAP2 mRNA and *FXN* mRNA levels in vitro in cell lines, suggesting that frataxin-deficiency-caused cellular iron deficiency may impact TFAP2 mRNA, leading to a further decrease in *FXN* mRNA [20].

Tumor suppressor protein p53 also controls the transcription of the *FXN* gene by binding to the p53-responsive element located upstream from the putative start site of transcription [67,68]. The inhibition of p53 function by pifithrin-α or the knockdown of p53 decreases the levels of *FXN* mRNA and protein. A recent study demonstrated that p53 directly binds to GAA-repeat-formed non-B DNA structures [69], though the outcome of this binding remains to be investigated.

Approaches that target the regions between SRF and TFAP2 binding sites significantly increase *FXN* gene expression. Transcription Activator-Like Effectors (TALE) are among them [70]. TALEs are DNA-binding proteins that contain repeated blocks of 34 amino acids that can be rearranged to target new DNA sequences [71]. TALE proteins can be fused with transcriptional activators such as VP64 or p300 to increase endogenous gene expression by the activation of transcription initiation of the target gene. In vitro in FRDA fibroblasts and in vivo in YG8R mice, nucleofecting plasmids expressing TALE-VP64s or delivering TALE-VP64s via the AAV vector induces the expression of the *FXN* gene and increases aconitase activity [70].

## 7. Iron

Frataxin plays a critical role in the synthesis of Fe-S clusters, which are protein cofactors that mediate redox reactions within the electron transport chain and in other pathways. The mitochondrial Fe-S cluster assembly complex comprises a few components: the cysteine desulfurase NFS1, its accessory protein ISD11, the assembly scaffold ISCU2, and frataxin itself [72,73]. Within this complex, frataxin is an activator of NFS1 activity and facilitates the transfer of sulfur to ISCU2 [74]. The interaction between frataxin and the Fe-S cluster assembly complex not only increases the efficiency of sulfur transfer but also supports the formation of a stable Fe-S cluster, which is essential for mitochondrial function. Therefore, in conditions such as FRDA, reduced frataxin levels compromise these Fe-S cluster biogenesis activities.

**Figure 1:**
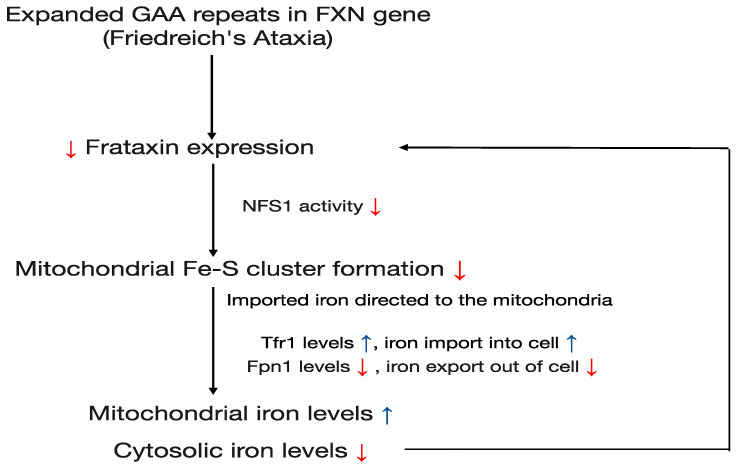
Mechanism linking iron metabolism and frataxin expression in the context of FRDA. Deficiency in frataxin leads to decreased NFS1 activity and reduced Fe-S cluster assembly. This results in elevated mitochondrial iron levels, which prompts an adaptive cellular response, characterized by upregulated Transferrin receptor 1 (TfR1) and downregulated Ferroportin 1 (Fpn1). This leads to enhanced iron import and reduced export, respectively. Because imported iron is preferentially directed to the mitochondria, cytosolic iron levels decline, leading to further frataxin decrease. In essence, this feedback loop perpetuates mitochondrial overload and frataxin deficiency in FRDA.

In patients with Friedreich’s ataxia (FRDA), there appears to be a positive feedback loop relating to iron levels and frataxin expression. Reduced levels of frataxin and disruptions in an Fe-S cluster assembly lead to iron accumulation within the mitochondria and a depletion of cytosolic iron [75,76]. This cytosolic depletion, in turn, leads to iron-dependent downregulation of *FXN* transcription [77]. As demonstrated in human cell lines and FRDA patient lymphoblasts and fibroblasts, iron chelator desferal (DFO) treatment decreases *FXN* mRNA and protein levels while ferric ammonium citrate, an iron salt, increases its levels. DFO treatment also reduces the expression of luciferase under the control of *FXN* promoter in vitro [77]. More specifically, frataxin deficiency results in the upregulation of transferrin receptor 1 (Tfr1) expression. Tfr1 binds to transferrin, a protein that transports iron in the blood, and allows for its cellular uptake. In FRDA, increased levels of Tfr1 on cell surfaces result in a greater influx of iron into the cells [78,79]. Rather than remaining in the cytosol, iron is preferentially directed towards the mitochondria, which senses a deficiency in iron due to the frataxin-related impairment of Fe-S cluster formation. Additionally, frataxin deficiency results in concurrent downregulation of ferroportin 1 (Fpn1), an iron-exporting protein [79]. The net result is a compounding problem of iron buildup within the mitochondria and iron depletion in the cytosol, leading to further downregulation of frataxin (Figure 1). Unknown is the precise mechanism via which iron regulates *FXN* gene transcription.

Strategies are developed to improve the distribution of iron between the cytosol and mitochondria in order to achieve a beneficial effect. Deferiprone, an iron chelator with a cell membrane crossing ability and low iron affinity, restores mitochondrial function in frataxin-deficient HEK293 cells [80], reduces ROS production, and improves calcium handling kinetics in an FRDA iPSC-derived cardiomyocyte model [81]. Deferiprone treatment, however, has mixed results in FRDA patients. While a low dose improves cardiac parameters, a high dose decreases frataxin levels and Fe-S enzyme activity, reflecting the iron depletion effect [82]. Therefore, iron chelator therapy should be used with caution.

## 8. Post-Transcriptional Regulation of *FXN* Gene Expression

Post-transcriptional regulation is the process of controlling the expression of genes at the RNA level through splicing, structural modification, or alteration of RNA stability. *FXN* is one of the genes whose expression can be altered by post-transcriptional regulation.

## 9. miRNAs

MicroRNAs (miRNAs) are short (18–23 nt) non-coding RNAs which bind predominantly to the 3′UTRs of complementary mRNAs and regulate their expression at the post-transcriptional level [83]. miRNAs are generally negative regulators of gene expression, yet they have occasionally been found to be positive regulators [84]. Differentially expressed miRNAs, both upregulated and downregulated, are found in FRDA patient cells including lymphoblasts, fibroblasts, periodontal ligament cells, and blood [85,86,87,88]. miRNA-224-5 and miRNA-886-3p are two upregulated miRNAs in FRDA patient cells that target the *FXN* transcript [85,86]. Overexpressing miRNA-224-5 decreases *FXN* mRNA and protein levels in Hela cells in vitro [85] while blocking miRNA-886-3p with anti-miRNA oligonucleotide raises *FXN* mRNA and proteins levels in FRDA fibroblasts. As the action of miRNA-886-3p also involves transcriptional control [86], further research is necessary to fully understand the mechanism underlying the regulation of miRNAs on *FXN* mRNA.

In addition to directly regulating *FXN* mRNA levels, miRNAs are also implicated in the pathogenesis of FRDA by regulating other genes such as brain-derived neurotrophic factor (BDNF). BDNF is an important regulator of neuronal growth, and reduced BDNF gene expression is found in patients with FRDA [85]. The gene transfer of *BDNF* into both primary neurons and a mouse model of FRDA impedes neurodegeneration [89]. miRNA-10a-5p negatively regulates *BDNF* mRNA by binding to its 3′UTRs [85]. In FRDA fibroblasts, miRNA-10a-5p is upregulated, while *BDNF* mRNA levels are decreased. Zinc-finger nuclease-mediated excision of the expanded GAA repeats corrects miRNA-10a-5p elevation, BDNF mRNA deficit, and FXN deficiency [85], highlighting the importance of miRNAs in the pathogenesis of FRDA and the possibility of miRNAs as FRDA treatment targets.

## 10. Post-Translational Regulation of Frataxin

Frataxin is produced in the cytosol as a precursor. Following synthesis, the frataxin precursor is imported into the mitochondria, where it undergoes mitochondrial processing peptidase (MPP)-mediated cleavage and maturation [90]. As a result, before becoming functional mature frataxin, frataxin post-translational regulation can happen at several stages and in several places.

## 11. Chaperones

GRP75, also known as mortalin or mtHsp70, is a multifunctional mitochondrial molecular chaperone of the heat shock protein family that is predominantly localized within the mitochondria, although it is also found in other cellular compartments [91,92]. GRP75 is involved in several physiological functions, such as protein folding, ISC protein synthesis, and cell survival, and is essential for maintaining cellular homeostasis and responding to stress [92,93,94,95]. Additionally, GRP75 participates in mitochondrial protein import. GRP75 is the core of the mitochondrial import motor complex that is required for translocation of most inner membrane or matrix-targeted proteins [96,97,98]. Whereas its C-terminal-peptide binding domain directly interacts with substrates, its N-terminal ATPase domain binds to ATP and hydrolyzes it to ADP. ATP hydrolysis not only provides energy for the membrane transport of the precursor polypeptides but also causes a conformational change in GRP75 that causes the precursor polypeptides to bind and to be released. GRP75 undergoes regulated cycling during the import process [98]. In yeast, the import and processing of the yeast frataxin homolog Yfh1p are impaired by mutations in the yeast GRP75 homologs SSC1 and SSQ1, which share 66% and 49% identity to GRP75, respectively. In contrast, GRP75 complements the function of yeast homologs in the maturation of Yfh1p [99,100,101]. GRP75 physically interacts with frataxin in human embryonic kidney 293 (HEK293) and COS7 cells, and knockdown of GRP75 decreases the level of frataxin in cancer cell lines [99].

GRP75 is a key post-translational regulator of both the amount and function of frataxin, controlling it both before and following mitochondrial import [102]. GRP75 overexpression raises the levels of precursor, intermediate, and mature frataxin in heterogeneous systems and rescues frataxin deficiency, ATP deficiency, and mitochondrial network defects in FRDA patient cells. GRP75 predominantly affects the frataxin precursor as the increase brought about by GRP75 overexpression on frataxin precursor is about nine times greater than that of the intermediate and mature forms, most likely reflecting its chaperone activity, which prevents the aggregation and degradation of frataxin precursor during its trafficking to mitochondria. The effect of GRP75 on mature frataxin is attributed to both increases in the pool of frataxin precursor and the interaction of GRP75 with frataxin and MPP, which results in the formation of a tertiary complex and enhanced accessibility and processing efficiency of frataxin by MPP [102]. Importantly, GRP75 overexpression has more prominent effects on clinically relevant missense frataxin variants including G130V, W168R, I154F, W155R, R165C, G137V, and I154F, which are found in compound heterozygote patients and, in general, lead to lower frataxin levels because of reduced protein stability and mitochondrial import. As GRP75 levels are decreased in multiple cell types of FRDA patients [102], restoring GRP75 might be effective in treating both typical FRDA patients with two GAA repeat expansions and compound heterozygous patients with point mutations.

Tumorous imaginal disc 1 (TID1), also called DnaJ homolog subfamily A member 3, mitochondrial (DNAJA3), is a novel binding partner of frataxin recently identified with a proteomic approach. TID1 is another member of the heat shock protein (Hsp) 40 family functioning as a cochaperone and regulatory component for Hsp70. TID1 interacts with the Hsp70 family of chaperone proteins via its distinctive J domain, a highly conserved tetrahelical region, which increases their ATPase activity for substrate binding [103,104,105,106,107]. TID1 also affects cell survival, proliferation, and responses to stress [108,109,110,111,112]. Acute and sub-acute frataxin deficiency results in elevated TID1 levels in multiple tissues including the cerebellum, skeletal muscle, and heart in a FRDA mouse model [113]. This elevation increases the frataxin precursor and decreases intermediate and mature forms in heterologous systems. In primary culture cells, TID1L and TID1S, two splice variants of TID1, exhibit differential roles in regulating frataxin levels. TID1S overexpression decreases mature frataxin, while TID1L overexpression has no effect. This could be ascribed to differences in their half-lives, protein interactome, and binding affinity to frataxin. The negative regulation of TID1S on frataxin is mediated by its last six amino acids (TID1S448-453) as a competing peptide generated from this sequence rescues frataxin deficiency and mitochondrial defects in FRDA patient-derived cells [113]. The small molecular weight and ease of modification offers the TID1S448-453 peptide a potential small molecule treatment option for FRDA.

## 12. Proteasome

Targeting the ubiquitin–proteasome system (UPS) is an increasingly more common method of small molecule therapeutics. The UPS is a major pathway in regulating the degradation of intracellular proteins, including that of frataxin. Proteins in the mitochondria are generally shielded from UPS-mediated degradation, but precursor frataxin levels are significantly reduced by the UPS before being imported into the mitochondrial matrix for maturation [114,115,116,117]. There is a possibility, however, that UPS-mediated degradation of frataxin may even occur on extramitochondrial mature frataxin [114]. Inhibition of the UPS causes the accumulation of both precursor and mature frataxin; therefore, a UPS-targeted strategy may be a beneficial therapy for treating FRDA patients.

Ubiquitination is a process involving the E1 ubiquitin-activating enzyme, E2 ubiquitin-conjugating enzyme, and E3 ubiquitin ligase. The E3 ligase recognizes the substrate to be ubiquitinated. The really interesting new gene (RING) finger protein 126 (RNF126) is the E3 ligase responsible for recognizing and interacting with the frataxin precursor, resulting in its ubiquitination and subsequent degradation. The inhibition of RNF126 leads to increased frataxin levels, making it a possible therapeutic target [115]. Since RNF126 has biological importance in quality control, an approach that does not disturb RNF126’s catalytic activity would be desirable.

Out of 13 potential lysine ubiquitination targets in frataxin, K^147^ is the main target because it is necessary and sufficient for frataxin ubiquitination through mono-ubiquitination. Loss of this ubiquitination site results in increased stability of frataxin due to its relative resistance to UPS-mediated degradation [114,116,117]. K^147^ is also the most conserved of frataxin’s 13 lysines [114]. K^147^ is a member of a well-defined, druggable cleft on the surface of frataxin. Small molecules called ubiquitin-competing molecules (UCMs) can be used to bind directly to the molecular cleft containing K^147^ to prohibit frataxin ubiquitination and degradation [114,116]. UCM interaction does not seem to alter frataxin function. This physical interaction leads to the accumulation of frataxin, as well as increased aconitase activity and ATP levels. Importantly, mature frataxin accumulation from UCM treatment reactivates Fe-S cluster biogenesis, supporting UCM as a potential FRDA therapeutic application [116].

Phosphorylation is another post-translational modification that interacts with ubiquitination to lead to frataxin degradation. Src tyrosine kinase phosphorylates frataxin on Y^118^, which then promotes frataxin ubiquitination. Inhibiting Src activity increases frataxin levels and rescues aconitase defects [117]. Therefore, Src kinase also has potential as a therapeutic target.

## 13. Mitochondrial Proteases

Mitochondrial proteases are the central regulators of mitochondrial proteostasis. In addition to their role as quality control enzymes that remove damaged proteins and prevent their possible deleterious accumulation, mitochondrial proteases regulate the half-life of proteins, play roles in mitochondrial protein maturation, such as MPP, and occasionally act as scaffolds without proteolytic activity [90,118,119]. The four functional groups of mitochondrial proteases include ATP-dependent peptidases, oliogo-peptidases, processing peptidases, and other mitochondrial peptidases [118]. Several mitochondrial proteases regulate the turnover of frataxin protein in addition to MPP. An siRNA screen in 293T cells targeting known mitochondrial proteases identified PITRM1, an ATP-dependent metalloprotease that breaks down post-cleavage mitochondrial transit peptides. Although the exact role of intermediate frataxin remains unknown, PITRM1 knockdown raises the amounts of intermediate frataxin in multiple cell lines and FRDA fibroblasts [120]. Other identified mitochondrial proteases in the same siRNA screen are SPG7/paraplegin and ClpP; however, their effects are not as strong as those of PITTRM1 [120].

The yeast mitochondrial Lon protease Pim1, which controls the turnover of oxidized proteins [121,122], also regulates frataxin. The deletion of Pim1 reverses the loss of Yfh1, the homolog of human FXN, in Erg29-deficient cells. A loss of function in the Erg29 gene (involved in the synthesis of ergosterol in yeast) increases the levels of 4′methyl sterol intermediates, leading to an iron-dependent oxidation of Yfh1 and subsequent decrease in Yfh1 levels [123]. Mitochondrial iron exporter (Mmt1) overexpression shields Yfh1 in ERG29-deficient cells from Pim1 mediated degradation [124]. In the same way that iron-treated FRDA fibroblasts exhibit a further reduction in frataxin levels, the Lon1 protease inhibitor 2-cyano3,12-dioxooleana-1,9-dien-28-oic acid methyl ester (CDDO–Me) counteracts this effect [124], suggesting that frataxin-deficiency-caused mitochondrial oxidative stress can in turn cause a further decrease in frataxin levels via Lon1 protease.

## 14. Autophagy

**Figure 2:**
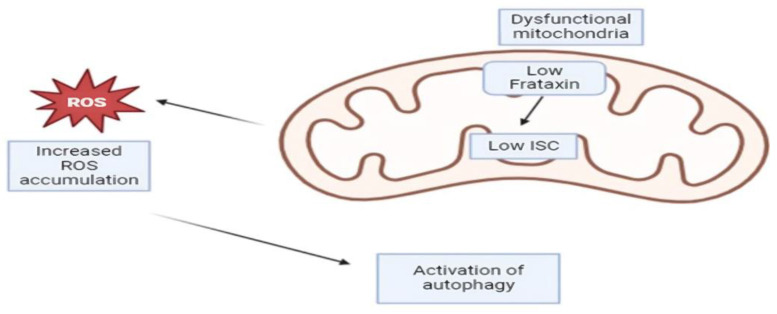
Autophagy activation. The decreased amount of frataxin leads to a dysregulated iron metabolism, a low amount of iron–sulfur clusters, and an upregulation of reactive oxygen species. This ultimately leads to the activation of autophagy.

Autophagy is a highly regulated mechanism that prevents the cell from self-destruction in a low-resource-nutrient environment. Autophagy is a multiple process involving the formation of autophagosomes, a double membrane-bound vesicle that engulfs a wide range of intracellular materials including misfolded proteins and damaged organelles, the fusion of autophagosome with lysosome, and the degradation of enclosed contents within the lysosome [125]. Along with the UPS, it is regarded as one of the main protein degradation systems. It is also thought of as a defense mechanism against ROS since it facilitates the breakdown of damaged proteins and cytosolic components [126]. Autophagy is often activated in FRDA in the aftermath of dysregulated iron and energy metabolism (Figure 2). Upregulated autophagic markers such as Atg3, p62, and FUNDC1 are observed in the hearts of the muscle creatine kinase conditional frataxin knockout mouse-MCK [127]. Such mice have positive iron staining in the hearts and markedly reduced cardiac function. In the nematode *Caenorhabditis* elegans model with frataxin silencing, autophagy is induced in a Parkin/*pdr-1*-, Pink/*pdr-1*-, and Bnip3/*dct-1*-dependent manner and involved in animal lifespan extension [128]. It was later found that Beclin and p53 are required for the induction of autophagy in *Caenorhabditis* elegans [129]. FRDA-patient-derived lymphoblasts also display increased autophagy, indicating an evolutionarily conserved response to reduced frataxin expression.

The role of autophagy has also been studied to determine its effect on precursor, intermediate, and mature frataxin levels. Transfecting siRNAs targeting ULK1 (UNC-51-like kinase 1), a regulator and a potential early initiator of autophagy, leads to a slight increase in intermediate but not mature frataxin in HEK293T cells [120]. Conversely, pharmacologically inducing autophagy with mTORC1/2 inhibitor INK128 diminishes intermediate frataxin signals without any effect on mature FXN levels, indicating that modulation of the autophagy pathway regulates intermediate frataxin but not mature FXN levels. Further understanding the role of intermediate frataxin and the mechanism underlying the regulation of autophagy on frataxin might provide a potential avenue for a therapeutic effect.

## 15. Conclusions

Further drugs are required for the treatment of FRDA, a neurological illness that progresses over time. One of the main therapeutic strategies for FRDA patients is the restoration of their frataxin levels. The knowledge of frataxin gene regulation at the transcriptional, post-transcriptional, and post-translational stages has advanced significantly. This has led to the identification of potential therapies including gene therapy, gene editing, and intervention to protein turnover (Table 1). However, there are still many unanswered questions and aspects of the control of the frataxin gene and its function that need to be explored. The answers to these queries may open new opportunities for treatment.

## Figures and Tables

**Table 1:** Treatment options related to *FXN* gene expression regulation.

*FXN* Gene Regulation Factors	Treatment Options
GAA repeat expansion	CRISPR-Cas9- or zinc finger nuclease-mediated removal of GAA repeat expansion
Transcription factors	Transcription Activator-Like Effectors (TALE) (TALE-VP64s)
Iron	Iron chelator-Deferiprone
miRNAs	Anti-miRNA oligonucleotide targeting miRNA-224-5 or miRNA-886-3p
Chaperones	GRP75 overexpression, TID1S448-453 peptide
Proteasome	Ubiquitin-competing molecules and Src tyrosine kinase inhibitor
Mitochondrial proteases	Inhibitors of PITRM1 and Lon1 protease
Autophagy	ULK1 inhibitor
